# Cutting-edge approaches to B-cell depletion in autoimmune diseases

**DOI:** 10.3389/fimmu.2024.1454747

**Published:** 2024-10-09

**Authors:** William H. Robinson, David Fiorentino, Lorinda Chung, Larry W. Moreland, Malavika Deodhar, Mary Beth Harler, Carrie Saulsbery, Rebecca Kunder

**Affiliations:** ^1^ Department of Medicine, Division of Immunology and Rheumatology, Stanford University, Stanford, CA, United States; ^2^ Palo Alto VA Health Care System, Palo Alto, CA, United States; ^3^ Division of Rheumatology, School of Medicine, University of Colorado Anschutz, Aurora, CO, United States; ^4^ IGM Biosciences, Mountain View, CA, United States

**Keywords:** autoimmune disease, B-cell depletion therapy, effector function-enhanced monoclonal antibodies, imvotamab, T-cell engager

## Abstract

B-cell depletion therapy (BCDT) has been employed to treat autoimmune disease for ~20 years. Immunoglobulin G1 (IgG1) monoclonal antibodies targeting CD20 and utilizing effector function (eg, antibody-dependent cellular cytotoxicity, complement-dependent cytotoxicity, antibody-dependent cellular phagocytosis) to eliminate B cells have historically been the predominant therapeutic approaches. More recently, diverse BCDT approaches targeting a variety of B-cell surface antigens have been developed for use in hematologic malignancies, including effector-function–enhanced monoclonal antibodies, chimeric antigen receptor T-cell (CAR-T) treatment, and bispecific T-cell engagers (TCEs). The latter category of antibodies employs CD3 engagement to augment the killing of target cells. Given the improvement in B-cell depletion observed with CAR-T and TCEs compared with conventional monospecific antibodies for treatment of hematologic malignancies and the recent case reports demonstrating therapeutic benefit of CAR-T in autoimmune disease, there is potential for these mechanisms to be effective for B-cell–mediated autoimmune disease. In this review, we discuss the various BCDTs that are being developed in autoimmune diseases, describing the molecule designs, depletion mechanisms, and potential advantages and disadvantages of each approach as they pertain to safety, efficacy, and patient experience. Additionally, recent advances and strategies with TCEs are presented to help broaden understanding of the potential for bispecific antibodies to safely and effectively engage T cells for deep B-cell depletion in autoimmune diseases.

## Introduction

The hallmark of autoimmune disease is recognition of self-antigens by the host immune system, leading to self-reactive T cells, autoantibodies, inflammation, and tissue damage (1). For decades, T cells were viewed as the major drivers of autoimmune disease, with B cells being only minor contributors. However, in the early 2000s anecdotal observations that anti-CD20 B-cell depletion therapy (BCDT) resulted in reduced disease measures in rheumatoid arthritis (RA) and other autoimmune diseases led to studies that transformed our appreciation of the role of B cells in multiple autoimmune diseases previously presumed to be mediated by T cells, including RA, multiple sclerosis (MS), systemic lupus erythematosus (SLE), and anti-neutrophil cytoplasmic antibodies associated vasculitis (1, 2). Because not all patients respond to treatment, and some BCDTs do not appear to fully deplete B-cell subsets that are important in autoimmune pathophysiology, including tissue-resident B cells or low-expressing CD20^+^ cells, it is now appreciated that deeper B-cell depletion from newer therapeutic approaches may provide increased efficacy in patients with autoimmune diseases (1). Approaches that offer 1) more extensive B-cell depletion beyond that achievable through conventional effector function mechanisms and 2) favorable safety and ease of administration appropriate for an outpatient setting are of particular interest for patients with chronic autoimmune diseases.

The array of immunotherapeutic BCDT approaches include monoclonal antibodies (mAbs) that target B-cell surface antigens, such as CD19 or CD20 (which are expressed on the surface of most cells of the B-cell lineage), B-cell specific chimeric antigen receptor T-cell (CAR-T) treatment, and, more recently, B-cell targeting bispecific T-cell engagers (TCEs) (1). Based on their efficacy and safety record in cancer, various BCDT approaches are now being repurposed for use in autoimmune diseases in which B cells play a central role (2).

In this review, we discuss the various BCDTs that are approved or are being developed for use in autoimmune disease. Whereas there is an abundance of recent literature describing cell therapy approaches for autoimmune diseases (3–5), the same is not true for TCEs. Therefore, additional focus on recent advances and strategies with TCEs is presented to help build a broader understanding of the potential for bispecific antibodies to safely and effectively engage T cells for deep B-cell depletion in autoimmune diseases.

## The role of B cells in autoimmune diseases

Autoimmune diseases, which result from abnormalities of the adaptive immune system, can be distinguished from autoinflammatory diseases, which are caused by hyperactivation of the innate immune system (6). B cells play an important role in a variety of autoimmune diseases (1), including major rheumatic autoimmune afflictions, such as SLE, RA, and antineutrophil cytoplasmic antibodies–associated vasculitis, as well as other autoimmune diseases, such as MS, myasthenia gravis, Graves’ disease, immunoglobulin (Ig) A nephropathy, immune thrombocytopenia, systemic sclerosis, and idiopathic inflammatory myopathies.

Both B and T cells are central components of the adaptive immune system (2). B cells are continuously generated throughout a person’s life in the bone marrow. B cells are primarily known as antibody-producing cells (7); however, in addition to antibody secretion, B cells have important functions, including antigen presentation and cytokine secretion, which can impact T cells and ultimately contribute to autoimmune pathology (2). Given their pleiotropic roles in autoimmune pathophysiology, B cells are attractive therapeutic targets for a range of autoimmune diseases.

**Figure 1:**
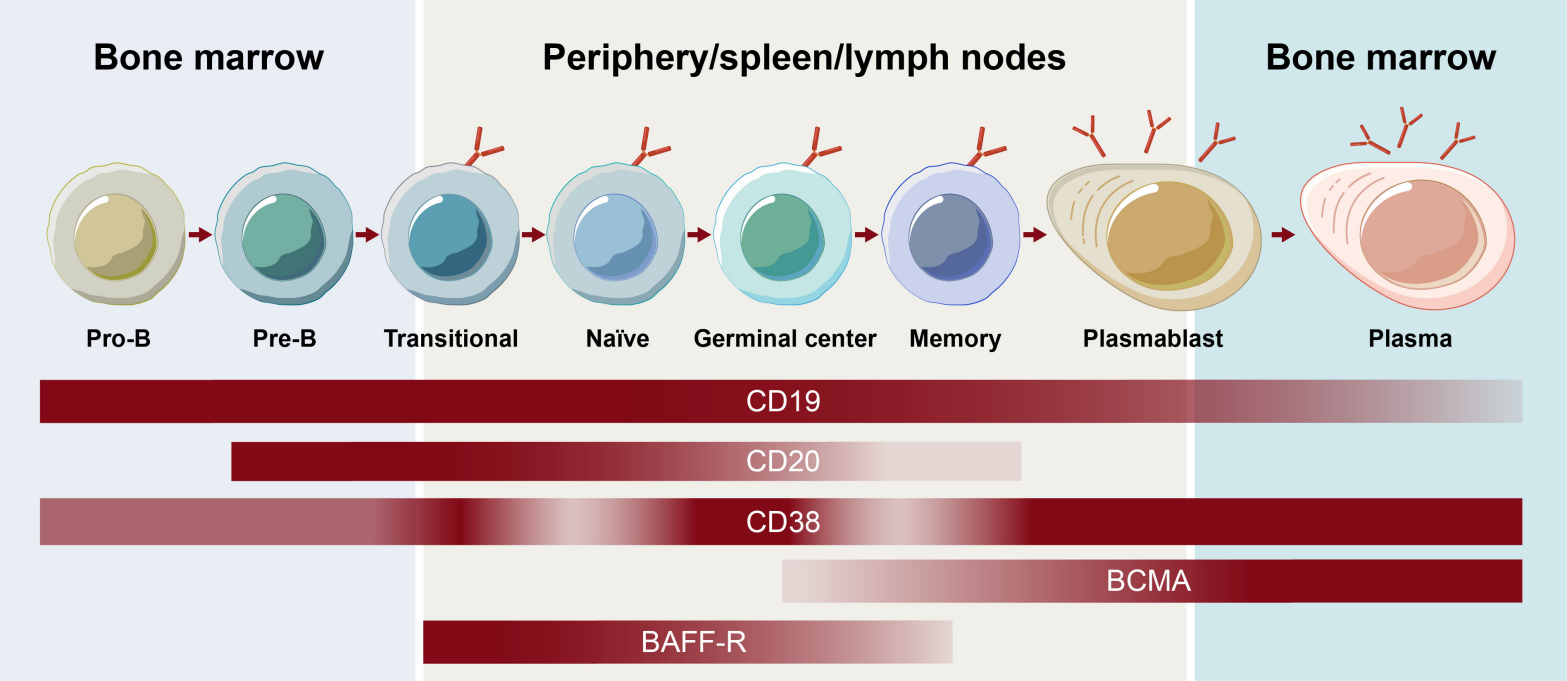
Receptor expression on B-cell subsets throughout B-cell development. BCMA, B-cell maturation antigen.

One of the earliest surface markers targeted to deplete B cells was CD20, which plays a role in B-cell receptor signaling, antigen response, and circulating memory B-cell development (8). Although more closely associated with B cells, CD20 expression has also been observed on T cells, possibly being acquired by T cells via trogocytosis from B cells (9). Like other surface markers, CD20 expression varies depending on the stage of B-cell development (**Figure 1**) (1). Although the majority of B cells express CD20, expression decreases during the plasmablast differentiation stage, and CD20 is not present on the surface of plasma cells or B-cell progenitors (10).

The contribution of the various subsets of B cells, plasmablasts, and plasma cells to autoimmune disease continues to be an active area of research. Memory B cells mature into short-lived plasmablast cells that, together with long-lived plasma cells, have been shown to be responsible for antibody secretion (2). Different surface antigens beyond CD20, including CD19, B-cell maturation antigen, CD38, and B-cell activating factor receptor, are expressed on unique subsets in the B cell lineage and play roles in the function and survival of B cells and/or plasma cells (**Figure 1**) (4). Assessing their effectiveness as BCDT targets continues to expand the understanding of the contribution of B-cell subsets in autoimmune disease and may ultimately allow for a more selective targeting of B-cell subsets to optimize the efficacy and safety of BCDT. Hence, an understanding of the breadth of current and emerging BCDT approaches is of high priority.

## B-cell depletion therapies

### Monoclonal antibodies

**Figure 2:**
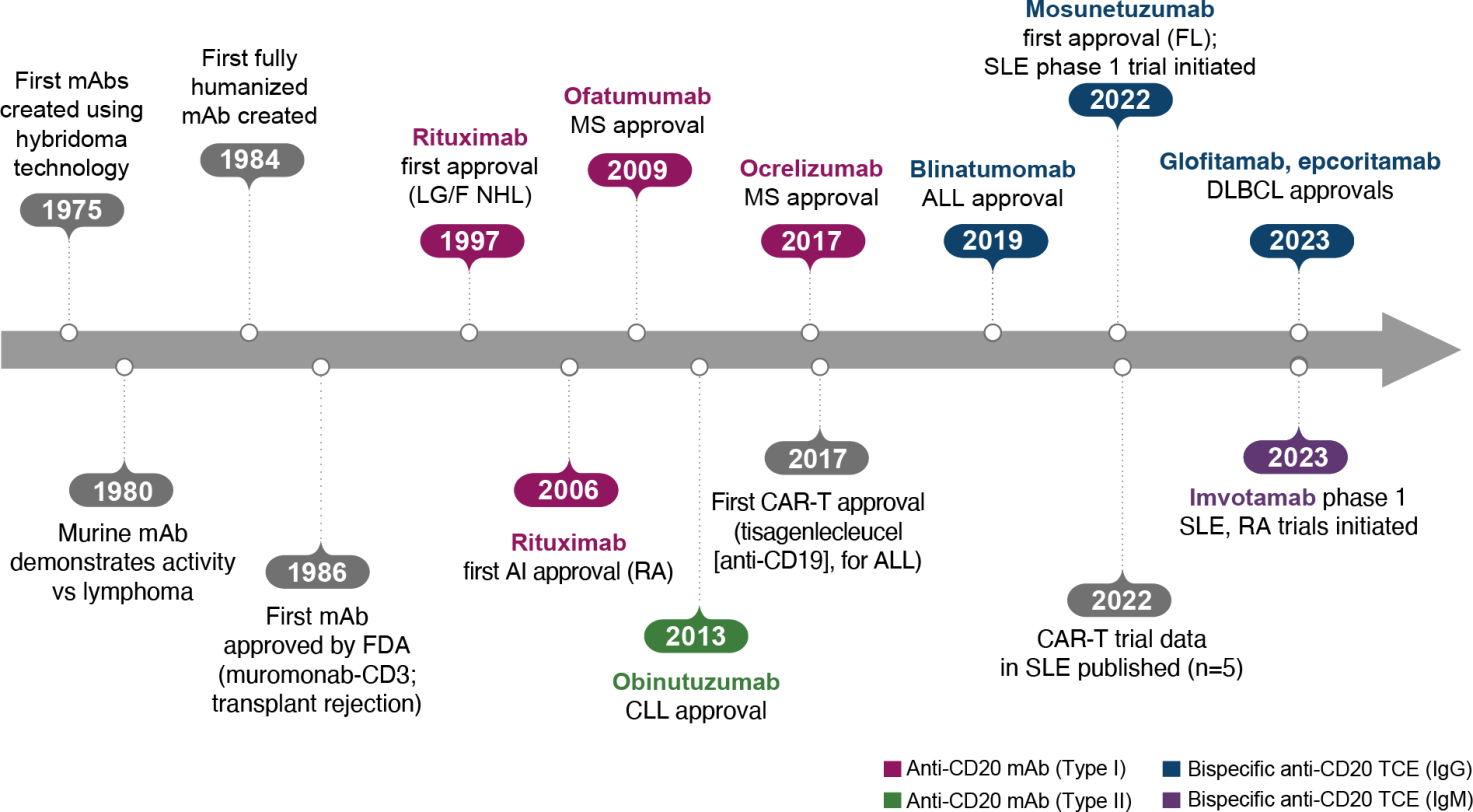
Key events in the BCDT timeline. ALL, acute lymphoblastic leukemia; BCDT, B-cell depletion therapy; CAR-T, chimeric antigen receptor T cell; CLL, chronic lymphocytic leukemia; DLBCL, diffuse large B-cell lymphoma; FDA, US Food and Drug Administration; FL, follicular lymphoma; Ig, immunoglobulin; mAb, monoclonal antibody; LG/F NHL, relapsed/refractory low-grade or follicular non-Hodgkin’s lymphoma; MS, multiple sclerosis; RA, rheumatoid arthritis; SLE, systemic lupus erythematosus; TCE, T-cell engager.

The era of BCDT began when the generation of mAbs with hybridomas became possible, leading to the creation of the first murine mAbs in 1975 (**Figure 2**) (10). The following decades of research demonstrated that these murine mAbs had activity against lymphoma and also later enabled the development of fully humanized antibodies. After approval of the first mAb, an anti-CD3 mAb (muromonab-CD3), for acute transplant rejection (1986), rituximab became the first anti-CD20 mAb, approved for relapsed/refractory low-grade or follicular non-Hodgkin’s lymphoma in 1997 and later for autoimmune diseases (initially for RA in 2006; **Figure 2**) (10).

Anti-CD20 mAbs have been shown to be active across a broad range of autoimmune conditions, albeit with varying degrees of efficacy (**Table 1**). Existing anti-CD20 mAbs can be categorized into type I, capable of translocation/clustering of CD20 into lipid rafts, and type II, which does not have this ability (11–13). Type I mAbs can cause cell death via 2 main mechanisms: antibody-dependent cellular cytotoxicity (ADCC), via signaling through activating Fc gamma receptors (FcγRs) expressed on natural killer cells, and complement-dependent cytotoxicity (CDC), which is accentuated by lipid raft clustering and occurs via binding of C1q, initiating activation of the classical complement pathway. Type I mAbs can also induce antibody-dependent cellular phagocytosis (ADCP), which involves recognition of opsonized target cells by phagocytes and direct cell death (11, 12). Type II anti-CD20 mAbs, which do not induce clustering of CD20 into lipid rafts, are less effective at CDC, but were optimized to induce direct killing of cells through lysosome-mediated cell death. Antibody engineering to enhance effector function including ADCC and ADCP has been utilized to enhance the efficacy of anti-CD20 mAbs (11, 12, 14).

**Table 1:** Comparison of traditional mAbs, bispecific anti-CD20 TCEs, and CAR-T therapies (1, 2, 12, 13, 15–17, 30, 31, 33–37, 42–48, 50, 52–54, 56, 57, 60–62, 67, 68).

	Traditional anti-CD20 mAbs			Bispecific anti-CD20 TCEs	Cellular therapy
	Type I		Type II	IgG, IgM*	CAR-T
	1st generation	2nd generation			
**Stage of Development in AI**	Approved in RA, AAV, PV	Approved in MS	No approved AI indications	No approved AI indications	No approved AI indications
**Example(s)**	Rituximab (mouse/human chimeric anti-CD20 mAb)	Ocrelizumab (humanized anti-CD20 mAb); ofatumumab (fully humanized anti-CD20 mAb)	Obinutuzumab (fully humanized anti-CD20 mAb)	Epcoritamab, glofitamab, mosunetuzumab (all CD20 × CD3 IgG Abs), imvotamab (CD20 X CD3 IgM Ab)	CD19 CAR-T
						**MOA**	ADCC (fucosylated, activating and inhibitory FcR), CDC, ADCP, limited apoptosis	ADCC (fucosylated, activating and inhibitory FcR), CDC, ADCP, limited apoptosis	Better ADCC vs type I mAbs (afucosylated Fc region), ADCP, CDC, apoptosis	IgG: TDCC, CD3 clustering IgM: TDCC, CDC	TDCC
**Other AI indications tested/under testing**	SLE: failed to show efficacy	N/A	SLE: some efficacy in case series of patients refractory to rituximab Lupus nephritis: efficacy suggested in phase 2; phase 3 trial ongoing	SLE: mosunetuzumab and imvotamab phase 1 trial ongoing RA: imvotamab phase 1 trial ongoing	SLE, SS, and IIM (antisynthetase syndrome): case series and small studies suggest efficacy
**Limitations**	Poor tissue depletion of B-cells; heterogeneity in clinical response	Poor tissue depletion of B-cells; heterogeneity in clinical response	heterogeneity in clinical response	IgG: Moderate CRS (all), ICANS (epcor) black box warnings; heterogeneity in clinical response IgM: unknown	High CRS and ICANS risk, need for lymphodepletion, heterogeneity in clinical response
**Dosing/Administration**	IV infusion; redose every 6-12 months, and disease recurs when redosing has stopped	SC injection monthly (ofatumumab); IV infusion every 6 months (ocrelizumab)	IV infusion	IV infusion (mosunetuzumab, glofitamab); SC injection (epcoritamab) IgM: IV infusion	Central line infusion; requires bespoke cell engineering & hospitalization

*Data from preclinical or clinical investigations of Imvotamab (CD20 X CD3 IgM based therapeutic) have been presented at conferences but not been published in peer-reviewed reports as of 8/9/2024.

AAV, anti-neutrophil cytoplasmic antibodies–associated vasculitis; Ab, antibody; ADCC, antibody-dependent cellular cytotoxicity; ADCP, antibody-dependent cellular phagocytosis; AI, autoimmunity; CAR-T, chimeric antigen receptor T cell; CDC, complement-dependent cytotoxicity; CRS, cytokine release syndrome; FcR, Fc receptor; ICANS, immune effector cell–associated neurotoxicity syndrome; Ig, immunoglobulin; IIM, idiopathic inflammatory myositis; IV, intravenous; mAb, monoclonal antibody; MOA, mechanism of action; MS, multiple sclerosis; PV, pemphigus vulgaris; RA, rheumatoid arthritis; SC, subcutaneous; SLE, systemic lupus erythematosus; SS, systemic sclerosis; TCE, T-cell engager; TDCC, T-cell–dependent cellular cytotoxicity. N/A, not applicable.

**Figure 3:**
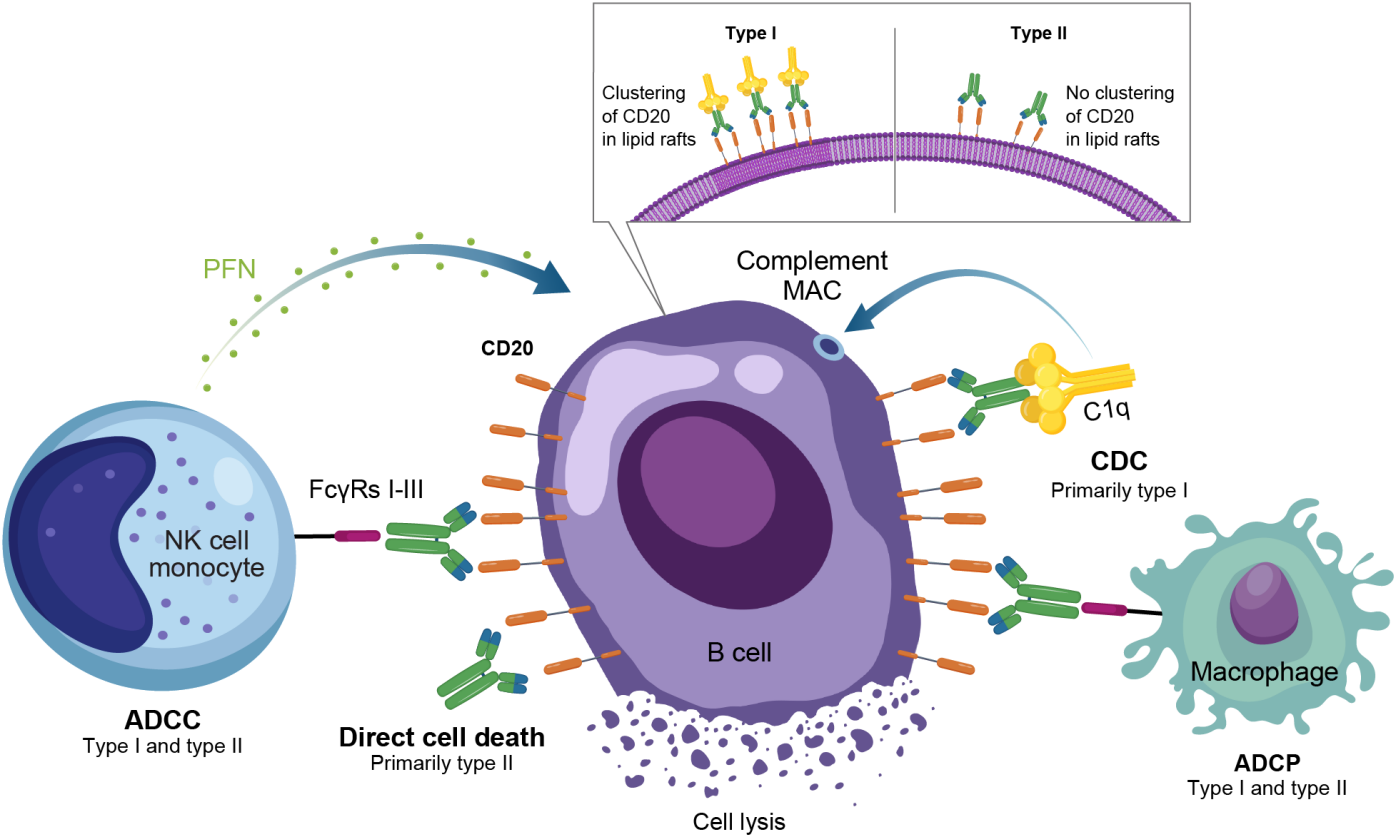
Overview of the MOA for anti-CD20 monoclonal antibodies in autoimmune disease. ADCC, antibody-dependent cellular cytotoxicity; ADCP, antibody-dependent cellular phagocytosis; CDC, complement-dependent cytotoxicity; FcγR, Fc gamma receptor; MAC, membrane attack complex; MOA, mechanism of action; NK, natural killer; PFN, perforin.

As a type I anti-CD20 mAb, the chimeric mouse/human rituximab relies on a combination of ADCC, CDC, ADCP, and direct cell death (10, 12, 13, 15) (**Figure 3**; **Table 1**). In addition to its use in lymphoma and leukemia, rituximab is approved for multiple autoimmune diseases (RA, granulomatosis with polyangiitis, microscopic polyangiitis, and pemphigus vulgaris) (16). However, rituximab was not found to be efficacious in patients with SLE (17) (**Table 1**). Even among diseases with demonstrated efficacy, not all patients respond to rituximab; anti-drug antibodies develop in approximately 30% of patients and low CD20-expressing B cells are not effectively depleted (18–21). Further, study of this type of anti-CD20 antibody in mice demonstrated the inability of effector function killing to deeply deplete B cells in the tissue (22). This may be due to insufficient effector cells and/or complement in the local tissue milieu, or the persistence of low expressing CD20^+^ cells, which cannot be effectively depleted through effector function due to the threshold expression requirement to activate these mechanisms. Intriguingly, in patients with RA, the lack of extensive depletion after rituximab was noted as a risk factor for relapse based on the level of circulating memory B cells (23–26). However, despite the lack of conclusive efficacy data, rituximab continues to be used in the management of SLE. It is also part of EULAR recommendations for SLE management (27), British Society of Rheumatology guideline for management of SLE (28), and American College of Rheumatology for treatment of lupus nephritis (29). To circumvent these and other limitations, next-generation type I anti-CD20 mAbs were developed, including ofatumumab (US Food and Drug Administration [FDA]–approved for MS in 2009) (30) and ocrelizumab (approved for MS in 2017; **Figure 2**) (31). Both ofatumumab and ocrelizumab are humanized mAbs, and as type I antibodies are capable of killing cells through the same mechanisms as rituximab, although ocrelizumab exhibits enhanced ADCC while ofatumumab is optimized for enhanced CDC (11, 12, 32). Ofatumumab is also administered subcutaneously, unlike rituximab and ocrelizumab, which are administered by intravenous infusion (16, 30, 31).

To further enhance ADCC, the fully humanized type II anti-CD20 mAb obinutuzumab was glycoengineered to be afucosylated at asparagine 297 in the Fc region (**Table 1**) (12, 13, 33–35). Because fucosylation is known to reduce binding affinity of IgG for the FcγR, and thus ADCC, afucosylation has led to higher binding affinity for obinutuzumab to FcγRIIIa; consequently, obinutuzumab activates FcγRIII more potently and has increased ADCC compared with type I anti-CD20 mAbs (12, 13, 33–35). ADCC and ADCP are the main mechanisms through which Type II antibodies cause B-cell depletion. Obinutuzumab is indicated for patients with cancer, including chronic lymphocytic leukemia following FDA approval in 2013, and is being evaluated in autoimmune diseases (**Figure 2**) (36). Obinutuzumab has demonstrated efficacy in a case series of patients with SLE who were refractory to rituximab (37), and also showed improved renal responses compared with placebo and was well tolerated in a randomized, phase 2 trial in patients with lupus nephritis (38); obinutuzumab is currently being developed for both SLE and lupus nephritis. Emerging studies may help elucidate the extent to which improved anti-CD20 monoclonal IgGs can address the limitations of traditional mainstays of treatment such as rituximab.

Though CD20 antibodies remain the focus of B-cell depletion therapies, inebilizumab, an anti-CD19 approved for treatment of a subset of patients with NMSOD was also shown to cause long-lasting B-cell depletion that correlated significantly with disease outcomes in a *post hoc* analysis (39). It is currently also being developed in management of IgG4- related disease. Additionally, antibodies like ianalumumab which target anti-B cell activating factor (BAFF-R) are also being developed to target autoimmune diseases like SLE and Sjogrens syndrome and have shown promising safety profiles in preliminary reports (40).

Ublituximab is another CD20- targeting monoclonal antibody that was recently approved for treatment of relapsing forms of multiple sclerosis. B-cell depletion via ADCC is described as the primary mechanism of action for ubilituximab (41). Though ublituximab has been tested in hematological cancers and NMSOD, no other autoimmune conditions are currently under development.

### Cell therapy

A range of CAR-T therapies are currently being evaluated in autoimmune diseases, including both mono- and bispecific approaches, to deplete distinct populations in the B-cell lineage (**Table 2**). Recent advances using anti-CD19 CAR-T therapy offer early but compelling evidence for substantial therapeutic benefit through deep B-cell depletion, as reflected by treatment-free remission in a small number of patients with severe, treatment-refractory autoimmune diseases (42–48). Potential safety concerns, the need for preconditioning, hospitalization, and logistic considerations of CAR-T production and administration are areas that currently limit CAR-T from being a widely accessible therapy for broad use in autoimmunity (49).

**Table 2:** Ongoing clinical trials with CAR-T in autoimmune diseases.

Antigen target	T-cell type	Phase	Indication(s)	Clinicaltrials.gov identifier	Trial sponsor
CD19	CAR-T (KYV-101)	2	MG	NCT06193889	Kyverna Therapeutics
BCMA	CAR-T (Descartes-08)	2	MG	NCT04146051	Cartesian Therapeutics
BCMA	CAR-T (Descartes-08)	2	SLE	NCT06038474	Cartesian Therapeutics
CD19	CAR-T (KYV-101)	1/2	SS	NCT06400303	Kyverna Therapeutics
CD19	CAR-T (YTB323)	1/2	SLE, LN	NCT05798117	Novartis
CD19	CAR-T (CABA-201)	1/2	IIM, dermatomyositis,antisynthetase syndrome, immune-mediated necrotizing myopathy	NCT06154252	Cabaletta Bio
CD19	CAR-T (CABA-201)	1/2	SLE, LN	NCT06121297	Cabaletta Bio
CD19	CAR-T (CABA-201)	1/2	SS	NCT06328777	Cabaletta Bio
CD19	CAR-T (MB-CART19.1)	1/2	SLE	NCT06189157	Miltenyi Biomedicine GmbH
CD19	Universal CAR-γδT	1/2	SLE	NCT06106893	Wuhan Union Hospital
CD19/CD20	CAR-T (MPT-514)	1/2	SLE	NCT06153095	ImmPACT Bio
CD19/BCMA/CD138/BAFF-R	CAR-T (4SCART)	1/2	B cell–related autoimmune diseases	NCT05459870	Shenzhen Genoimmune Medical Institute
CD19	CAR-T (CC-97540)	1	SLE, IIM, SS	NCT05869955	Bristol Myers Squibb
CD19	CAR-T (CC-97540)	1	MS	NCT06220201	Bristol Myers Squibb
CD19	CAR-T (KYV-101)	1	IIM, SS, SLE, AAV	NCT06152172	Kyverna Therapeutics
CD19	CAR-T (KYV-101)	1	LN	NCT05938725	Kyverna Therapeutics
CD19	CAR-T (KYV-101)	1	Dermatomyositis	NCT06298019	Stanford University
CD19	CAR-T (KYV-101)	1	MS	NCT06138132	Stanford University
CD19	CAR-T (KYV-101)	1	IIM, SS, SLE, AAV	NCT06152172	University of Pennsylvania
CD19	CAR-T	1	SLE	NCT03030976	Shanghai GeneChem Co
CD19	CAR-T (Relma-cel)	1	SLE	NCT05765006	Shanghai Ming Ju Biotechnology Co
CD19	CAR-T	1	MG	NCT05828225	Zhejiang University
CD19	CAR-T	1	Neuromyelitis optica spectrum disorder	NCT05828212	Zhejiang University
CD19	CAR-T	1	SLE, Sjögren’s syndrome, SS, dermatomyositis, AAV	NCT06056921	Chongqing Precision Biotech Co
CD19	CAR-T (RD06-04)	1	SLE	NCT06310811	Wuhan Union Hospital
CD19	CAR-T (obe-cel)	1	SLE	NCT06333483	Autolus Limited
CD19	Universal CAR-T (SC291)	1	SLE, AAV, polyangiitis	NCT06294236	Sana Biotechnology
CD19/BCMA	CAR-T	1	SLE	NCT05474885	iCell Gene Therapeutics
CD19/BCMA	CAR-T (GC012F)	1	SLE	NCT05846347	Zhejiang University
CD20/BCMA	CAR-T (C-CAR168)	1	SLE, immune-mediated necrotizing myopathy, neuromyelitis optica spectrum disorder, MS	NCT06249438	RenJi Hospital
DSG3	CAR-T ((DSG3-CAART))	1	Mucosal-dominant PV	NCT04422912	Cabaletta Bio
MuSK	CAR-T	1	MG (anti-MuSK antibody-positive)	NCT05451212	Cabaletta Bio
CD19	CAR-T (CNCT19)	Early phase 1	SLE	NCT05930314	Peking Union Medical College Hospital
CD19	CAR-T (CNCT19)	Early phase 1	SLE	NCT06316791	Juventas Cell Therapy Ltd.
CD19/BCMA	CAR-T (GC012F)	Early phase 1	SLE	NCT05858684	Renji Hospital
CD19/BCMA	CAR-T	Early phase 1	SLE	NCT05030779	Zhejiang University
CD19/BCMA	CAR-T	Early phase 1	Immune nephritis, LN	NCT05085418	Zhejiang University
CD19/BCMA	CAR-T	Early phase 1	SS	NCT05085444	Zhejiang University
CD19/BCMA	CAR-T	Early phase 1	Sjögren’s syndrome	NCT05085431	Zhejiang University
CD19/BCMA	CAR-T	Early phase 1	Immune nephritis, LN	NCT05085418	Zhejiang University
CD19/BCMA	CAR-T	Early phase 1	POEMS syndrome, amyloidosis, AIHA, vasculitis	NCT05263817	Zhejiang University
BCMA	CAR-T (CT103A)	Early phase 1	Neuromyelitis optica spectrum disorder, MG, chronic inflammatory demyelinating polyradiculoneuropathy, immune-mediated necrotizing myopathy	NCT04561557	Tongji Hospital
CD7	CAR-T	Early phase 1	Crohn’s disease, UC, dermatomyositis, Still disease	NCT05239702	Zhejiang University
CD19	Universal CAR-T (BRL-301)	NA	SLE, Sjögren’s syndrome, SS, IIM, AAV, antiphospholipid syndrome	NCT05859997	Bioray Laboratories
BCMA	CAR-T (PRG-1801)	NA	LN, AAV	NCT06277427	Tongji Hospital

The information in the table was obtained from clinicaltrials.gov, last accessed on May 17, 2024.

AAV, antineutrophil cytoplasmic antibodies–associated vasculitis; AIHA, autoimmune hemolytic anemia; BCMA, B-cell maturation antigen; CAAR, chimeric autoantibody receptor; CAR-T, chimeric antigen receptor T cell; CAAR-T, chimeric autoantibody receptor T cell; IIM, idiopathic inflammatory myopathy; LN, lupus nephritis; MG, myasthenia gravis; MuSK, muscle-specific tyrosine kinase; NA, not applicable; POEMS, polyneuropathy, organomegaly, endocrinopathy, monoclonal gammopathy, and skin changes; PV, pemphigus vulgaris; SLE, systemic lupus erythematosus; SS, systemic sclerosis; UC, ulcerative colitis.

**Figure 4:**
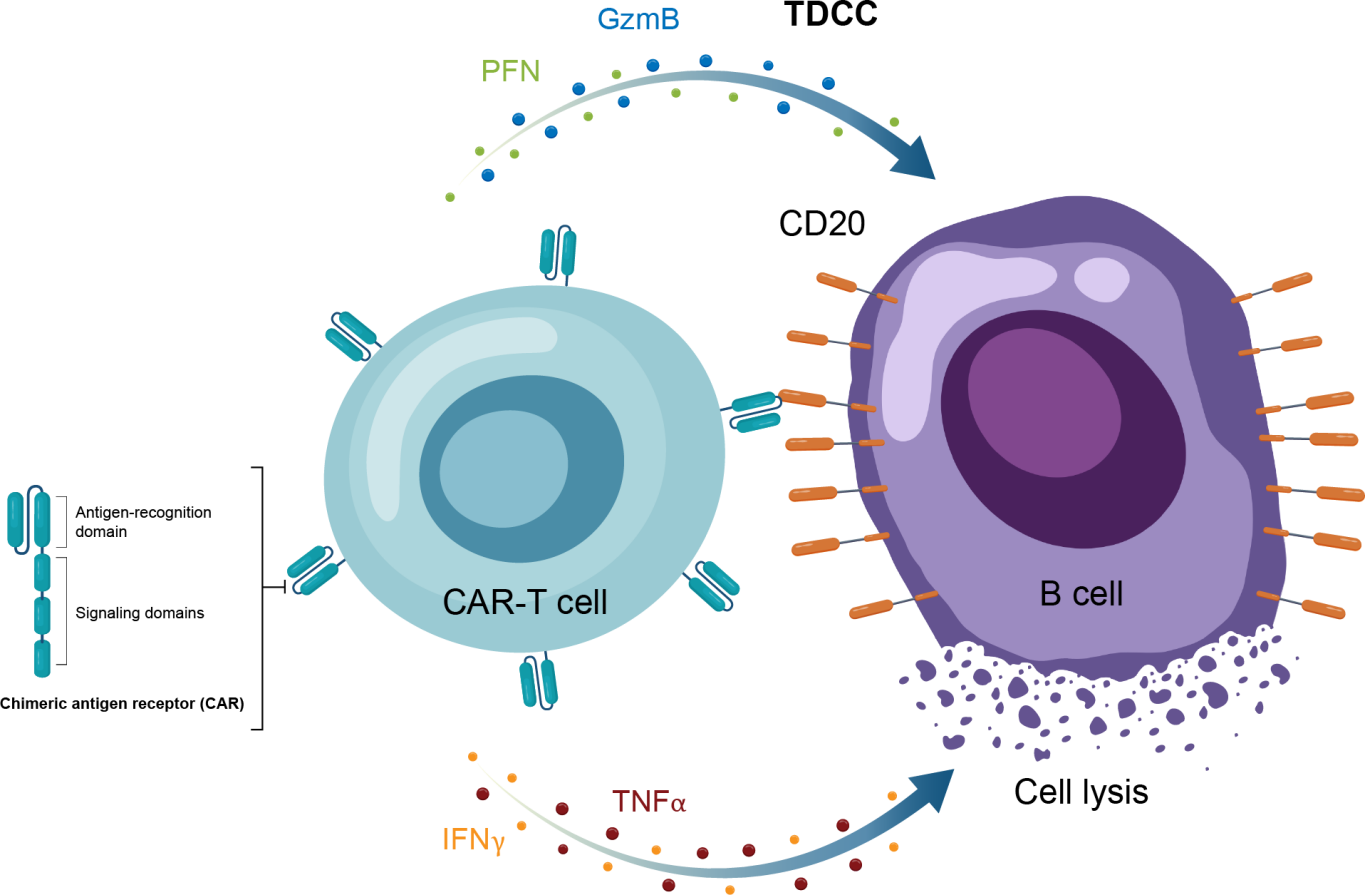
Overview of the MOA for CAR-T therapy in in autoimmune disease. CAR-T, chimeric antigen receptor T cell; GzmB, granzyme B; IFNγ, interferon gamma; MOA, mechanism of action; PFN, perforin; TNFα, tumor necrosis factor alpha.

Autologous CAR-T therapy, initially developed for hematologic malignancies, requires genetic modification of an individual patient’s T cells to express specifically tailored synthetic receptors targeting cancer cells (50). Allogenic “off-the-shelf” CAR-T approaches, derived from healthy donors rather than individual patients, are also under development (51). CAR-Ts are activated through binding of the tumor-associated antigen to the chimeric antigen receptor (CAR). This results in T-cell secretion of cytokines and cytotoxins, such as granzyme and perforin, which ultimately kill the target tumor cells through T-cell dependent cellular cytotoxicity (**Table 1**, **Figure 4**) (52). The first CAR-T therapy approval in 2017 was for the CD19-targeted tisagenlecleucel in B cell precursor acute lymphoblastic leukemia (**Figure 2**) (53).

Anti-CD19 CAR-T therapy in patients with various autoimmune conditions was first reported in 2021, including a small series of patients with treatment-refractory SLE, antisynthetase syndrome, and systemic sclerosis (42–48). Follow-up (median, 15 months) in these patients demonstrated impressive and durable clinical responses in the majority of patients treated with CAR-T therapy (54). These preliminary findings have resulted in initiation of a number of clinical trials focused on treatment of moderate-to-severe SLE and an array of other autoimmune diseases (**Table 2**).

Attendant to the promising efficacy with CAR-T therapy are several notable limitations, including a need for lymphodepletion and hospitalization, high costs of therapy, and serious safety risks. Two of the most important risks of administration are cytokine release syndrome (CRS) and immune effector cell–associated neurotoxicity syndrome (ICANS) (50, 53). These toxicities can often be managed with tocilizumab (with or without steroids) but can also be life-threatening. The long-term safety profile of CAR-T therapy, including the potential risk for secondary T-cell malignancy that is being investigated by the FDA (55), is yet to be fully characterized. In addition, the high financial burden of CAR-T therapies presents a significant limitation for many patients. Thus, despite compelling evidence of efficacy in a small number of patients, the safety, logistical, and cost drawbacks of CAR-T therapies have led to an unmet need for efficacious and well tolerated treatments for patients with autoimmune diseases who may require lifelong treatment. Advances in CAR-T from autologous to allogeneic cells may help to reduce some of the logistical concerns, as may other approaches, such as use of RNA instead of DNA-based CAR and the use of natural killer cells in CAR-based cell therapy.

### Immune-cell engagers

**Figure 5:**
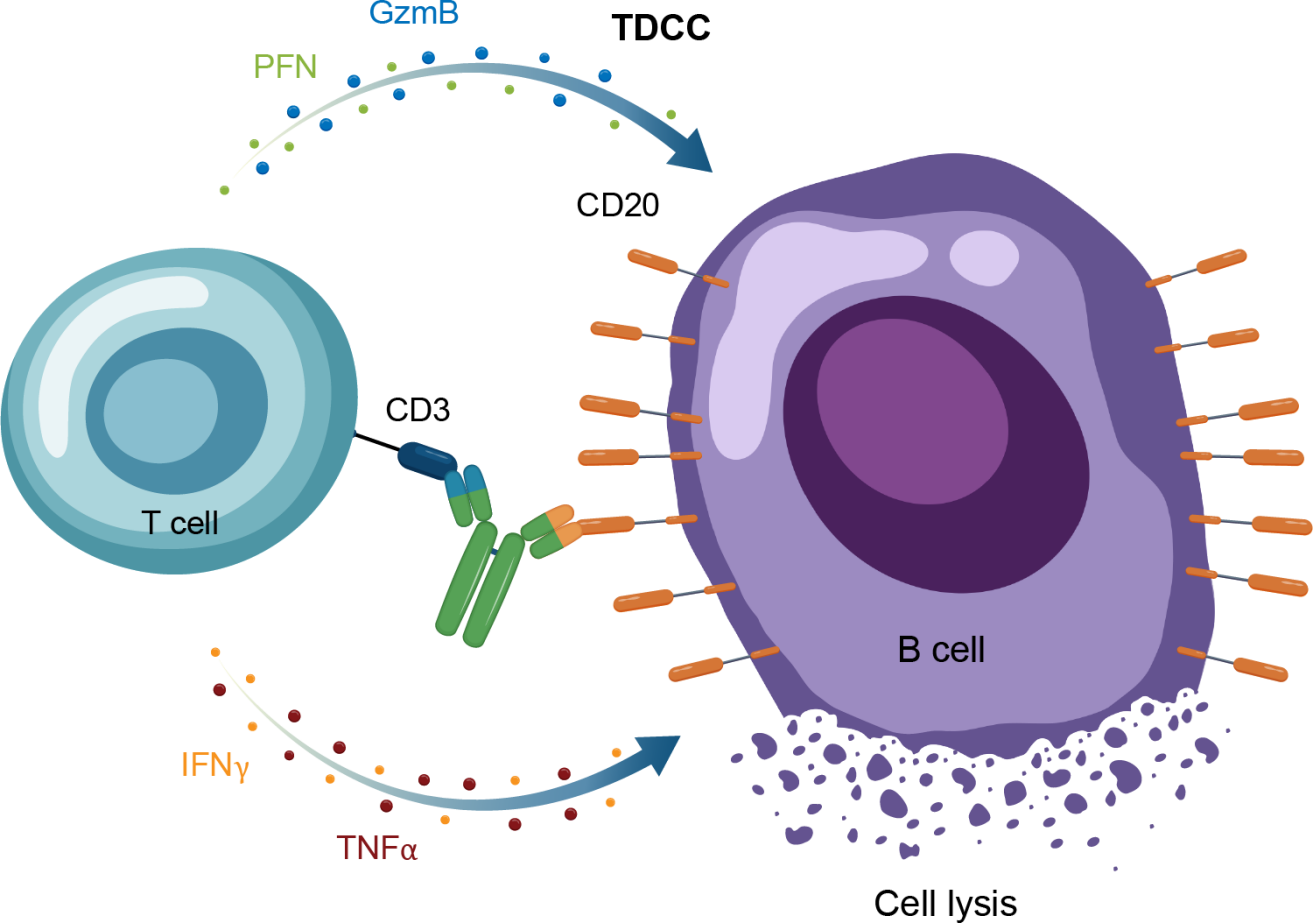
Overview of the MOA for IgG TCEs in autoimmune disease. GzmB, granzyme B; Ig, immunoglobulin; IFNγ, interferon gamma; MOA, mechanism of action; PFN, perforin; TDCC, T-cell dependent cellular cytotoxicity; TNFα, tumor necrosis factor alpha.

A recent therapeutic advance in oncology, which is also being applied to autoimmune diseases, is use of bispecific immune cell engagers, which redirect effector cells to tumor cells (56, 57). T-cell engaging antibodies have binding specificity for target cells and effector T-cells, bridging the 2 by targeting specific antigens and an activating T-cell surface molecule resulting in cytotoxic cell death (58). In the context of oncology, the target cell is the tumor cell and the target antigen is a tumor-specific antigen, whereas in autoimmune disease, the target cell is a pathogenic cell expressing a target antigen or “address” that brings the T cell to that pathogenic cell to kill it. Structurally, these molecules have a binding arm directed to a tumor-associated antigen (eg, CD20) and at least 1 other arm targeting an activating receptor on an immune effector cell, with the most common approach to date being CD3 for T cells (58). For TCEs, the activated T cells then form an immune synapse with the target cells, with the TCE acting as the bridge. This leads to the release of cytokines, perforin and additional granzymes, causing target cell lysis but also carrying with it a risk of cytokine release syndrome and neurotoxicity (**Figure 5**) (57).

The CD19 × CD3–targeting agent blinatumomab was the first B-cell–depleting TCE approved by the FDA in 2014 (for B-cell precursor acute lymphoblastic leukemia; **Figure 2**) (59), and additional B-cell depleting TCEs, including those targeting CD20 × CD3, were approved thereafter (mosunetuzumab for follicular lymphoma in 2022; glofitamab, and epcoritamab for diffuse large B-cell lymphoma in 2023) (60–62). These bispecific CD20 × CD3 IgG molecules, which utilize CD3 clustering to generate strong T-cell activation and as a result T-cell dependent cellular cytotoxicity as the mechanism of action for cell killing (**Table 1**; **Figure 5**) (57), have black box warnings for CRS in their label (60–62), with instructions to utilize step-up dosing to reduce the incidence and severity of CRS. Epcoritamab also has a black box warning pertaining to ICANS risk, and is the only approved TCE that can be administered subcutaneously (61). Although these safety risks have seemingly limited the scope of use for TCEs outside of oncology, with no approved indications or available data in autoimmune diseases, a phase 1 trial with mosunetuzumab in patients with SLE was initiated in 2022, with primary data expected later this year (ClinicalTrials.gov Identifier: NCT05155345; **Table 3**). B-cell depletion and clinical improvement were recently reported using the CD19 × CD3 bispecific blinatumomab (administered as a continuous infusion for 5 days per cycle) to treat 6 patients with RA (2 cycles) (63) and a patient with rapidly progressive severe systemic sclerosis (4 cycles) (64).

**Table 3:** Ongoing clinical trials with TCEs in autoimmune diseases.

Drug	Specificity	Ab isotype	Indication	Clinicaltrials.gov identifier	Phase	Trial sponsor
Imvotamab	CD20 × CD3	IgM	RA	NCT06087406	1	IGM Biosciences
Imvotamab	CD20 × CD3	IgM	SLE	NCT06041568	1	IGM Biosciences
Mosunetuzumab	CD20 × CD3	IgG	SLE	NCT05155345	1	Hoffmann-La Roche
RO7507062	CD19 × CD3	unknown	SLE	NCT05835986	1	Hoffmann-La Roche

Ab, antibody; Ig, immunoglobulin; RA, rheumatoid arthritis; SLE, systemic lupus erythematosus; TCE, T-cell engager.

**Figure 6:**
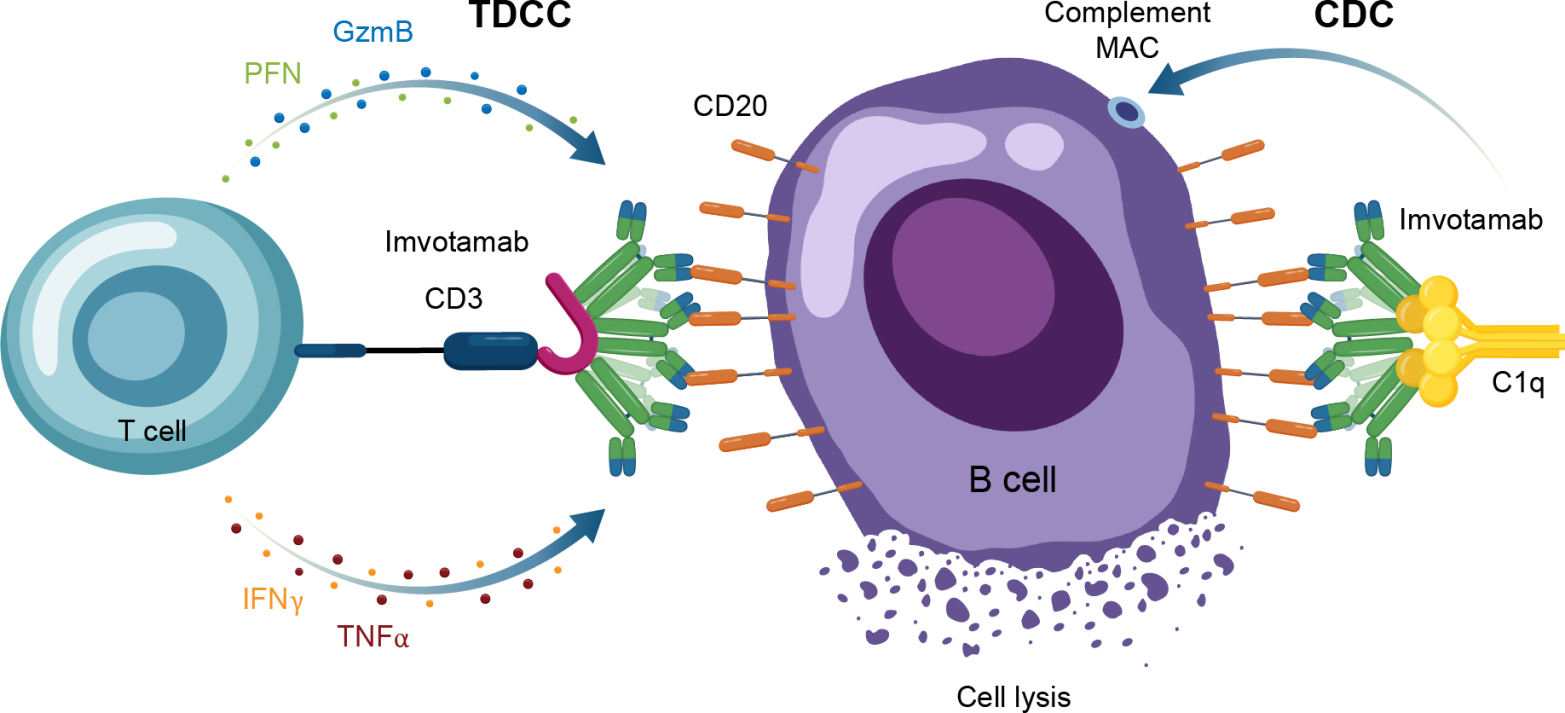
Overview of the MOA for IgM TCEs in autoimmune disease. CAR-T, chimeric antigen receptor T cell; CDC, complement-dependent cytotoxicity; GzmB, granzyme B; IFNγ, interferon gamma; MAC, membrane attack complex; MOA, mechanism of action; PFN, perforin; TDCC, T-cell dependent cellular cytotoxicity; TNFα, tumor necrosis factor alpha.

Another TCE in development for use in autoimmune conditions is the CD20 × CD3 IgM molecule imvotamab (IGM-2323; **Table 1**). The structure of imvotamab consists of 10 high-affinity IgG CD20-binding domains attached to a pentameric IgM scaffold, with 1 anti-CD3 single-chain fragment variable attached to the J chain (65, 66). Although traditional mAbs, and approved TCEs, are typically built on an IgG framework, the IgM structure may be uniquely suited to use in a bispecific TCE due to the larger size and 10 high-affinity CD20 target binding sites in the IgM format.This results in IgM-based TCEs having high target avidity and potentially more controlled levels of T-cell activation as compared to corresponding IgG-based TCEs. Theoretically, this high avidity would enable imvotamab to cause depletion of low target-expressing cells, including activated memory B cells, using a combination of T-cell–dependent cellular cytotoxicity and CDC (**Figure 6**), with potentially less CD3 clustering that is exhibited by traditional IgG TCEs (**Table 1**). Preclinical data, as well as phase 1 data in patients with advanced B-cell malignancies, have suggested the potential for B-cell depletion in tissue and in low CD20^+^ expressors, without evidence of high levels of CRS and other adverse events (65, 66). Phase 1 clinical trials with imvotamab have been initiated in RA (ClinicalTrials.gov Identifier: NCT06087406) and SLE (ClinicalTrials.gov Identifier: NCT06041568), with data availability projected for 2025 for both trials (**Table 3**, **Figure 2**); an additional trial in myositis is planned. Based on the data obtained from these studies, the potential for an IgM-based TCE in patients with autoimmune diseases can be more thoroughly assessed.

## Discussion and future perspectives

As the threads of the immune system are increasingly intertwining malignancy and autoimmune disease, leveraging oncology drug discovery and development for the treatment of autoimmunity is increasingly fruitful. The recent CAR-T data in patients with severe autoimmune diseases offer a view into the potential of deep BCDT. Off-the-shelf approaches, such as allogeneic cell therapy and TCEs, may have the potential to deliver better clinical outcomes than achievable with current therapies, but without many of the logistical, financial, and safety considerations associated with autologous CAR-T. Clinical studies are ongoing to characterize risks and benefits specifically in autoimmune conditions. Data from ongoing trials with TCEs in patients with autoimmune conditions (with mosunetuzuamb [SLE] and imvotamab [RA, SLE]), may shed more light on the early promise of these therapeutic agents in autoimmunity. Additionally, potential beyond repurposed TCEs from oncology exists to target other immune or nonimmune cell types (eg, fibroblasts), to utilize multispecific TCEs for tissue-specific delivery, and to engage alternative effector cells (eg, NK cells). The therapeutic landscape continues to evolve as we fully harness novel technologies to address specific cell types and pathways responsible for autoimmunity and to ultimately bring relief to those suffering from autoimmune diseases.

## Glossary

AAV: antineutrophil cytoplasmic antibodies–associated vasculitis
ADCC: antibody-dependent cellular cytotoxicity
ADCP: antibody-dependent cellular phagocytosis
ANCA: anti-neutrophil cytoplasmic antibodies
AI: autoimmunity
AIHA: autoimmune hemolytic anemia
ALL: acute lymphoblastic leukemia
BCDT: B-cell depletion therapy
BCMA: B-cell maturation antigen
CAAR: chimeric autoantibody receptor
CAR: chimeric antigen receptor
CAR-T: chimeric antigen receptor
CLL: chronic lymphocytic leukemia
CDC: complement-dependent cytotoxicity
CRS: cytokine release syndrome
DLBCL: diffuse large B-cell lymphoma
FcγR: Fc gamma receptor
FDA: US Food and Drug Administration
FL: follicular lymphoma
GzmB: granzyme B
ICANS: immune effector cell–associated neurotoxicity syndrome
IFNγ: interferon gamma
Ig: immunoglobulin
IgG1: immunoglobulin G1
IIM: idiopathic inflammatory myopathy
IV: intravenous
LN: lupus nephritis
mAb: monoclonal antibody
MAC: membrane attack complex MG, myasthenia gravis
MOA: mechanism of action
MS: multiple sclerosis
MuSK: muscle-specific tyrosine kinase
NK: natural killer
NMOSD: neuromyelitis optica spectrum disorder
PFN: perforin
POEMS: polyneuropathy, organomegaly, endocrinopathy, monoclonal gammopathy, and skin changes
PV: pemphigus vulgaris
RA: rheumatoid arthritis
SC: subcutaneous
SLE: systemic lupus erythematosus
SS: systemic sclerosis
TCE: T-cell engager
TDCC: T-cell–dependent cellular cytotoxicity
TNFα: tumor necrosis factor alpha
UC: ulcerative colitis
